# Improving Indoor Pedestrian Dead Reckoning for Smartphones under Magnetic Interference Using Deep Learning

**DOI:** 10.3390/s23239348

**Published:** 2023-11-23

**Authors:** Ping Zhu, Xuexiang Yu, Yuchen Han, Xingxing Xiao, Yu Liu

**Affiliations:** 1 School of Geospatial Information and Geomatics Engineering, Anhui University of Science and Technology, Huainan 232001, China; ping3583@163.com; 2 Coal Industry Engineering Research Center of Mining Area Environmental and Disaster Cooperative Monitoring, Anhui University of Science and Technology, Huainan 232001, China; 3 School of Earth and Environment, Anhui University of Science and Technology, Huainan 232001, China; 4 School of Geomatics and Urban Spatial Informatics, Beijing University of Civil Engineering and Architecture, Beijing 102616, China; 5 College of Geography and Environmental Sciences, Zhejiang Normal University, Jinhua 321000, China

**Keywords:** magnetic interference, pedestrian dead reckoning, indoor positioning, convolutional neural network, support vector machine, unscented Kalman filter

## Abstract

As micro-electro-mechanical systems (MEMS) technology continues its rapid ascent, a growing array of smart devices are integrating lightweight, compact, and cost-efficient magnetometers and inertial sensors, paving the way for advanced human motion analysis. However, sensors housed within smartphones frequently grapple with the detrimental effects of magnetic interference on heading estimation, resulting in diminished accuracy. To counteract this challenge, this study introduces a method that synergistically employs convolutional neural networks (CNNs) and support vector machines (SVMs) for adept interference detection. Utilizing a CNN, we automatically extract profound features from single-step pedestrian motion data that are then channeled into an SVM for interference detection. Based on these insights, we formulate heading estimation strategies aptly suited for scenarios both devoid of and subjected to magnetic interference. Empirical assessments underscore our method’s prowess, boasting an impressive interference detection accuracy of 99.38%. In indoor environments influenced by such magnetic disturbances, evaluations conducted along square and equilateral triangle trajectories revealed single-step heading absolute error averages of 2.1891° and 1.5805°, with positioning errors averaging 0.7565 m and 0.3856 m, respectively. These results lucidly attest to the robustness of our proposed approach in enhancing indoor pedestrian positioning accuracy in the face of magnetic interferences.

## 1. Introduction

Today, an increasing number of intelligent services require location-based services as a prerequisite, such as the autonomous driving of vehicles, emergency rescue, and intelligent coal mining. In open, unobstructed outdoor scenes, satellite-based navigation systems can provide relatively accurate positioning. However, when in narrow, obstructed indoor environments, electromagnetic signals from satellites are reflected, diffracted, and attenuated, making them unsuitable for high-precision indoor positioning [1]. How to solve the “last mile” of positioning has gradually become a hotspot in current positioning research.

### 1.1. Related Work

Indoor positioning based on fingerprints builds a location fingerprint map using previously collected wireless signal strength (or other types of sensor data), and then compares the collected data with this map to determine the location [2,3,4,5]. This method requires the establishment and maintenance of a lot of additional infrastructure, such as Bluetooth [6] and WiFi positioning [7], which require multiple anchor nodes or base stations and collect and store a large amount of training fingerprint data. Fingerprint-based indoor positioning has strong environmental dependence and requires a fingerprint database to be built for a specific environment, consuming a lot of material and human resources and increasing time costs [8]. In emergencies like earthquakes or fires, the destruction of infrastructure and changes in the indoor environment render the positioning system non-functional [9], greatly limiting its feasibility and flexibility in practical applications.

In contrast, pedestrian dead reckoning (PDR) is an algorithm that facilitates positioning without the need for any external infrastructure or signals [10]. This method directly leverages sensor data to offer real-time or near-real-time location insights, ensuring robust privacy protection as it operates independently of external devices. It is characterized by its autonomy and minimal susceptibility to external environmental influences [11]. PDR, when implemented on smartphones, finds extensive applications in indoor positioning, virtual reality, and human health monitoring and tracking. Furthermore, it can be seamlessly integrated with GPS, Bluetooth, and WiFi sensors, enabling multi-sensor fusion-based positioning, thereby enhancing both the precision and stability of location estimates [12].

Heading estimation, as one of the core steps of the PDR algorithm, is often the primary reason for drift in positioning results. Methods for heading estimation include those based on magnetometer signals [13,14], those based on gyroscope angular velocity [12,15,16,17], and those based on the fusion of magnetometer signals and angular velocity [18,19]. Combining magnetic heading angles with gyroscope heading angles is a prevalent strategy aimed at enhancing heading estimation accuracy. Magnetometers offer an absolute heading reference based on the Earth’s magnetic field, while gyroscopes are adept at capturing rapid angular changes. However, each comes with its set of limitations. Magnetometers are susceptible to local magnetic field disturbances, such as those from electronic devices or steel reinforcements within buildings, whereas gyroscopes might accumulate errors, leading to prolonged heading drifts [20]. By fusing data from these two sensors, their shortcomings can be mutually compensated to some extent [21]. For instance, when the magnetometer experiences transient interference, the gyroscope data can serve as a stable heading reference. Conversely, when the gyroscope begins to accumulate errors, the magnetometer can offer an absolute heading correction.

Existing sensor fusion-based heading estimation nclude the Kalman filter (KF), extended Kalman filter (EKF), unscented Kalman filter (UKF), particle filter (PF), and complementary filter (CF). The simplest KF fusion of gyroscope and magnetometer methods for integrated heading solutions was proposed in [22]. However, pedestrian movement is mostly non-linear and is challenging to adapt to the non-linear systems for extended experiments. Both the EKF and the UKF are non-linear extensions of the KF, maintaining sensitivity and adaptability to various non-linear changes. The study in [23] employed a two-step EKF algorithm to estimate the inertial measurement unit (IMU)’s orientation, yielding accurate direction. However, the EKF encounters divergence problems stemming from the linear model derived from the Jacobian matrix. The reference [24] proposed the UKF to address the divergence problem in IMU attitude estimation. The study in [25] introduced a UKF heading estimation method that fuses accelerometers, magnetometers, and gyroscopes, reducing heading errors in non-linear systems. The research in [26] mitigates interferences introduced by surrounding environments but performs poorly with non-linear systems. Reference [27] put forth a new quaternion-based complementary filter; this filter avoids the roll and pitch components of direction affected by magnetic disturbances, yet the heading remains significantly influenced. Experimental results indicate that compared to other sensor fusion techniques, UKF technology possesses higher heading accuracy [21].

However, for low-cost magnetometers, mitigating the effects of magnetic interference poses a challenge. During indoor pedestrian movement, the reliability of heading estimation based on magnetometers diminishes due to frequent interference from nearby ferrous materials and other pedestrians carrying phones. In certain scenarios, additional strategies or sensors are still required to further enhance the accuracy and stability of heading estimation. The study in [28] analyzed the impact of local magnetic disturbances on the accuracy of magnetic and inertial sensors. Reference [29] introduced a quaternion-based indirect Kalman filter to avoid magnetic interference affecting pitch and roll estimation, yet it does not specifically address the adverse impact on yaw estimation. Reference [30] utilizes a CNN to extract pedestrian walking sensor feature information and employs LSTM to predict pedestrian step length. However, LSTM has the drawback of computational complexity. Reference [31] employs a CNN to recognize landmarks in real-time within PDR positioning trajectories, calibrating the accumulated PDR error. However, the Softmax function classification has issues with precision. Reference [32] adopts the SVM algorithm to classify 19 different pedestrian activities for indoor navigation positioning. However, redundant data can impact the classification results. Reference [33] introduces a method based on manual feature extraction, followed by an SVM model to recognize local magnetic field interference during walking. This approach aims to optimize the KF fusion algorithm’s heading estimation accuracy when affected by local magnetic interference. However, the feature extraction method is relatively complex.

### 1.2. Contributions and Structure

This paper employs the UKF heading fusion algorithm, effectively reducing heading errors in non-linear systems. To further optimize, we harness CNNs to automatically extract features, which are then fed into the SVM algorithm for magnetic interference detection. This approach not only streamlines the manual feature extraction process but also bolsters the algorithm’s automation capabilities. As a result, the accuracy of pedestrian dead reckoning is significantly enhanced in situations where the local magnetic field is interfered with.

Therefore, the development of an algorithm that can automatically extract features to detect magnetic interference, reducing unnecessary feature redundancy, becomes crucial for heading estimation in smartphones during motion. The primary contributions of this paper are as follows:

(1) In this study, based on sensor data during walking, we developed a deep-learning algorithm using CNNs, aimed at extracting profound features from pedestrian movements and effectively minimizing feature redundancy.
(2) Furthermore, we introduced a deep-learning model that integrates both CNNs and SVMs, tailored specifically for detecting local magnetic field disturbances during walking. In addition, we conducted a comparative analysis of the performance differences in heading estimation between standalone CNNs or SVMs and the combined CNN–SVM model proposed in this paper.
(3) Depending on the presence of magnetic interference, we proposed two distinct heading fusion strategies. In environments free from local magnetic interference, we employed the UKF to merge data from gyroscopes and magnetometers. Conversely, in environments with local magnetic disturbances, we determined the final heading based on the previous moment’s ultimate heading, combined with the gyroscope’s heading data.

In Experiment 1, which utilized a square trajectory, the detection accuracy for local magnetic interference achieved 99.38%. The heading estimation had an average absolute error of merely 2.1891°, and the mean positioning error stood at 0.7865 m. Meanwhile, in Experiment 2, following a triangular trajectory, the detection accuracy for local magnetic interference reached 99.16%, with the heading estimation’s average absolute error being 1.5805° and the mean positioning error at 0.3856 m. These results underscore that our deep-learning-based model adeptly identifies distortions in the local magnetic field across varied trajectories, thereby enhancing indoor pedestrian positioning accuracy in the presence of magnetic disturbances.

The rest of this paper is organized as follows: Section 2 introduces the theoretical basis of our algorithm, the magnetic interference detection model, heading estimation methods, and accuracy evaluation standards. In Section 3, we discuss the data collection and preprocessing steps for our experiments and then analyze the experimental results. Finally, in Section 4, we present our conclusions and future work directions.

## 2. Materials and Methods

### 2.1. PDR Algorithm Principle

**Figure 1:**
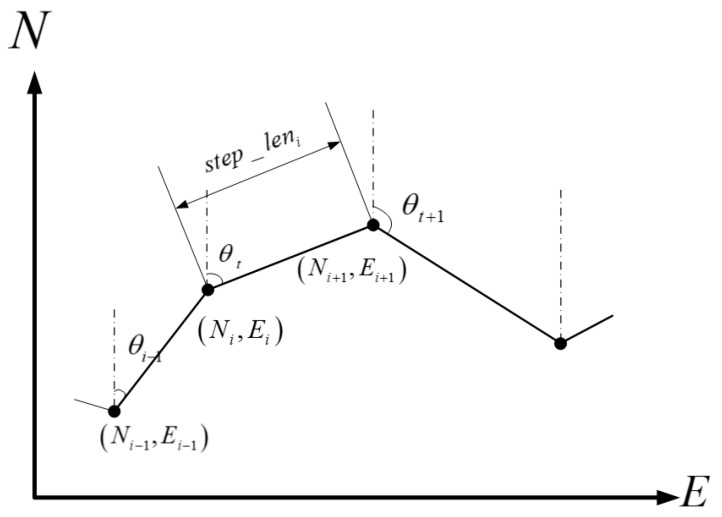
Schematic diagram of the calculation of the pedestrian track.

PDR, when utilizing smartphone inertial and magnetic sensors, provides a straightforward and cost-effective solution for indoor positioning. The algorithm primarily encompasses three essential steps: step detection, step-length estimation, and heading determination [34]. The PDR positioning principle is illustrated in Figure 1.

The motion equation can be expressed using Equation (1):

(1) $$\begin{array}{l} N_{i}=N_{0}+\sum_{n=1}^{i}\mathrm{step}\_len_{n}\times \cos\theta_{n}\\ E_{i}=E_{0}+\sum_{n=1}^{i}\mathrm{step}\_len_{n}\times \sin\theta_{n} \end{array}$$

In the equation: $N_{0}$ and $E_{0}$ represent the initial plane position; $\mathrm{step}\_len_{n}$ represents the step length of the nth step; and $\theta_{n}$ represents the heading angle of the *n*th step.

### 2.2. Algorithm Framework of This Study

**Figure 2:**
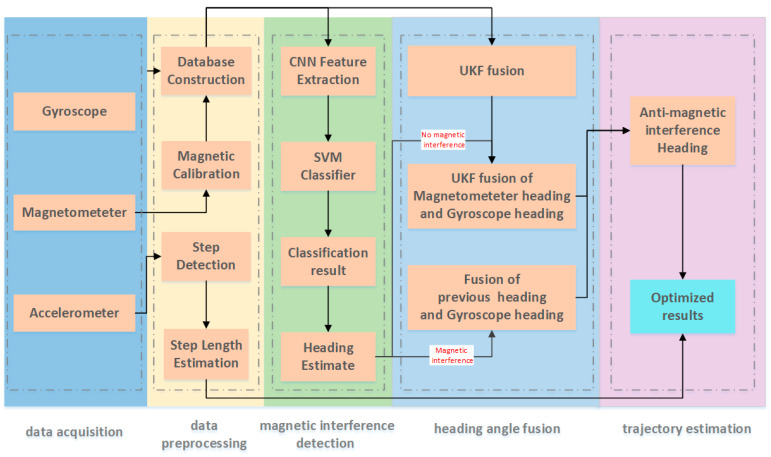
Framework of the heading estimation algorithm based on CNN–SVM for magnetic interference detection.

To better estimate pedestrian trajectory in indoor environments and enhance the heading estimation accuracy using smartphones, this study introduces the algorithm framework depicted in Figure 2. This framework is divided into five steps: data collection, data preprocessing, magnetic interference detection, heading angle fusion, and trajectory estimation. Data preprocessing encompasses step frequency detection, step-length estimation using acceleration data, magnetometer calibration with magnetic data, and CNN–SVM model database construction from smartphone inertial and magnetic data.

### 2.3. Data Collection

**Figure 3:**
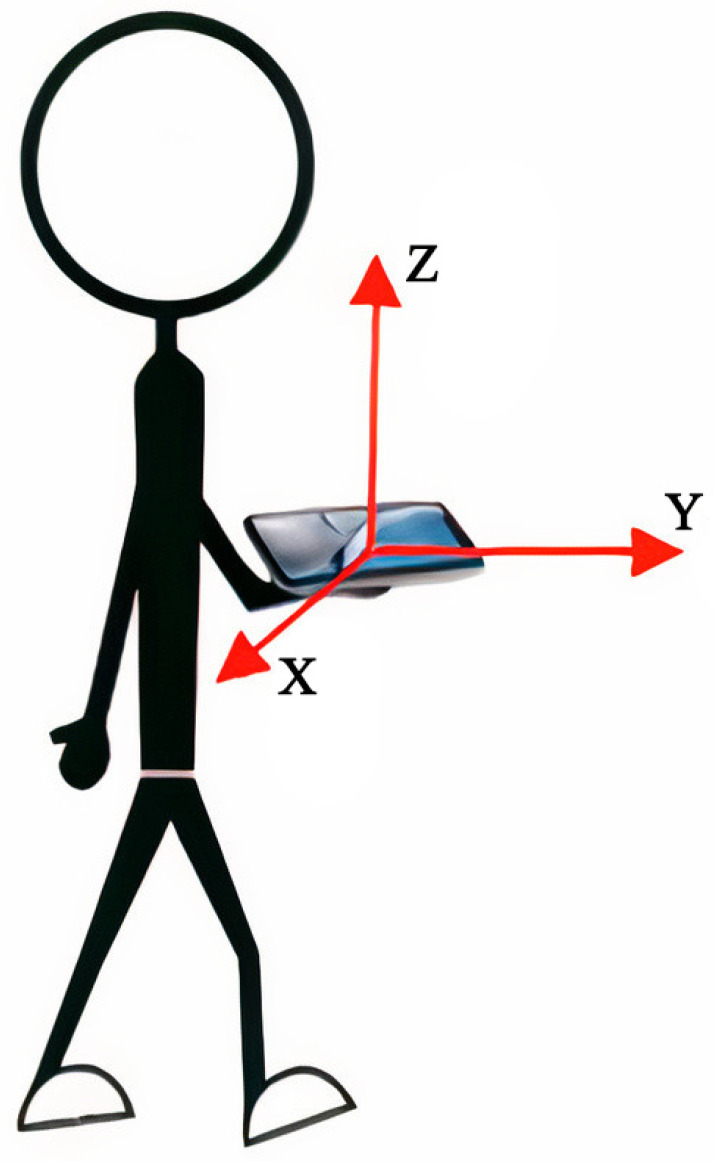
Illustration of a pedestrian holding a smartphone while walking.

**Figure 4:**
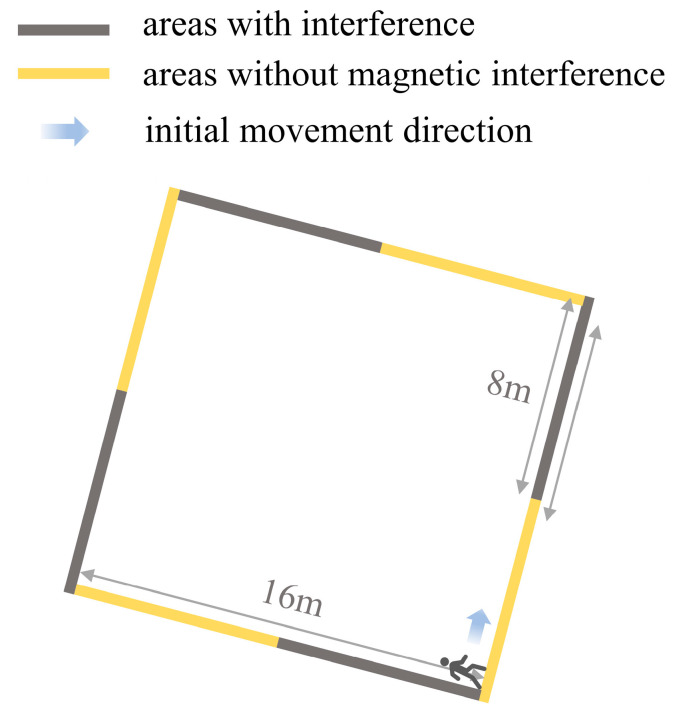
Predefined square trajectory.

This study utilized an iPhone Xr, manufactured by Apple Inc., Cupertino, CA, USA, to collect tri-axis acceleration, gyroscope, and magnetometer data during pedestrian movement, recording at a frequency of 100 Hz. As illustrated in Figure 3, the participant held the smartphone level in front of their body and walked at a consistent pace. For the experiment involving local magnetic interference, the participant utilized a common iron object from daily life—a key—as a source of interference with the local magnetic field. Under these conditions, the participant, holding both the smartphone and the key, captured data for 480 uniform steps. In contrast, for the experiment without magnetic interference, only the smartphone was used to record the same 480 steps. To ensure the precision of our model evaluation, the experimental area was meticulously gridded into squares measuring 0.8 m × 0.8 m each. The participant walked along a square path with a perimeter of 16 m (comprising 20 grids), ensuring that each step spanned exactly 0.8 m, with the tip of the foot landing on the grid intersections. During data collection, as depicted in Figure 4, the participant initially walked 10 steps without any magnetic interference. They then paused and slowly brought the key closer to the smartphone to introduce magnetic interference. Once the magnetic field stabilized, the participant proceeded to walk another 10 steps while holding the key. This process was repeated until two full laps, as shown in Figure 4, were completed. The yellow sections represent areas without magnetic interference, while the black sections indicate areas with interference, totaling 80 steps per lap.

### 2.4. Data Preprocessing

Step Detection: Walking involves a rhythmic lifting and falling of the feet, propelling the body forward and causing periodic variations in acceleration. However, the raw acceleration data often contains substantial high-frequency noise and numerous spurious peak values, making direct step detecting challenging [35]. In this study, we computed the composite acceleration magnitude from the three-axis linear acceleration using Equation (2):

(2) $$a=\sqrt{a_{x}{}^{2}+a_{y}{}^{2}+a_{z}{}^{2}}-g$$

In the formula: a represents the composite acceleration magnitude; $a_{x}$, $a_{y}$ and $a_{z}$ represent the accelerations of the accelerometer in the x, y, and z axes, respectively; and *g* represents the local gravitational acceleration magnitude.

After computing the composite acceleration, we introduced a low-pass filter. This filter effectively removed the pseudo-peaks present in the composite acceleration, ensuring that they did not interfere with the stride frequency detection process. Following this filtering stage, we employed the threshold-peak detection method [36]. This technique precisely identifies the peaks in the acceleration data, allowing us to determine the total number of steps taken during walking.

Stride length estimation: In the realm of pedestrian navigation, determining a person’s walking distance is crucial and is achieved by summing up individual stride lengths. While numerous techniques exist for estimating stride length [37], we opted for the widely accepted Weinberg method in this study [38]. This method elucidates an empirical relationship, specifically a fourth-degree correlation, between stride length and the vertical acceleration range. The mathematical representation of this relationship is detailed in Equation (3).

(3) $$\mathrm{Weinberg}=K\times \sqrt[{4}]{a_{\max}-a_{\min}}$$

In the formula: K represents the constant parameters of the stride length model; $a_{\max}$ and $a_{\min}$ are the maximum and minimum values in the acceleration sequence of each step, respectively.

Magnetometer Calibration: It is a well-accepted fact that, under optimal conditions, when a magnetometer undergoes sufficient rotation, its geomagnetic values will map onto a sphere centered at the origin (0, 0, 0). In this study, prior to data collection, a smartphone was manually maneuvered in a “figure eight” pattern in the indoor experimental environment. This ensured that the device experienced comprehensive motion across all axes (X, Y, and Z), facilitating the collection of magnetic field data from various orientations. The magnetic field data from the indoor environment were then gathered, and the magnetometer data was fitted using the least squares method, yielding the calibration matrix A and the offset vector b specific to the environment. The magnetometer data in both the training and testing datasets were calibrated according to Equation (4).

(4) F=(D−b)*A

In the formula: D represents the initial magnetometer data, *F* represents the magnetometer data after calibration.

**Figure 5:**
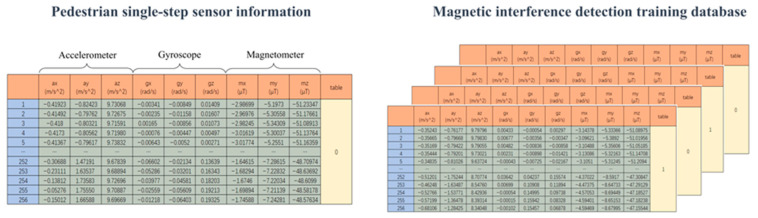
Schematic diagram of the pedestrian magnetic interference detection database.

Data Segmentation: Utilizing the results from step frequency detection, we processed the gathered data from the accelerometer, gyroscope, and magnetometer. Each step’s peak served as a reference, from which we extracted 127 sample points before the peak and 128 following it, resulting in a 256 × 9 matrix per step. Based on the presence or absence of magnetic interference, we curated the training data and associated labels. The data structure is illustrated in Figure 5.

### 2.5. Model Construction

#### 2.5.1. CNN Principle

The convolutional neural network (CNN) is a type of deep-learning model frequently employed for analyzing visual imagery [39]. As pedestrians move, the IMU sensors in smartphones generate vast amounts of data. While this data encapsulates information about the pedestrian’s movement, it also contains some noise. CNNs, through their multi-layered convolution and pooling operations, can discern hierarchical features from the data. By extracting more abstract and representative features from the noisy raw data, CNNs enhance the robustness of classification algorithms.

**Figure 6:**
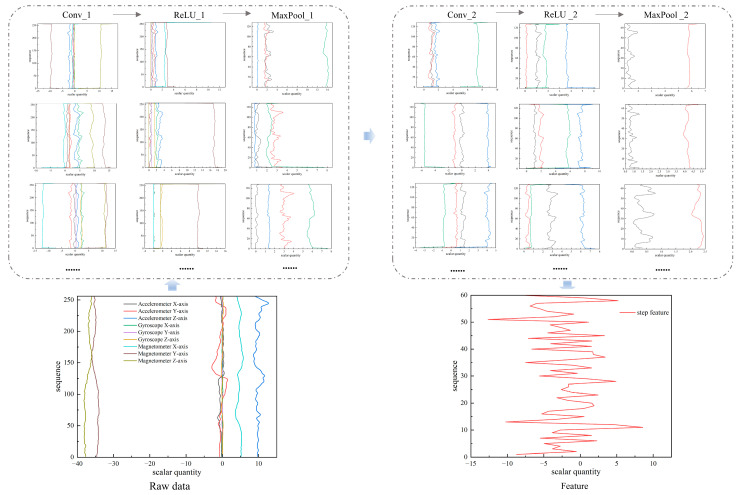
Architecture and information workflow of the CNN model for single-step feature extraction.

Considering the local features and spatial relationships among the nine-axis sensors in smartphones, this study treats the data from a single step of the nine-axis sensor as a two-dimensional input. Two-dimensional convolutional kernels slide over the input data to extract features. The non-linear activation function, ReLU, is then employed, enabling the network to learn and capture intricate features from the input data. Subsequently, max-pooling operations are used for downsampling, reducing the spatial dimensions of the data and thereby decreasing computational complexity. Within the CNN, after undergoing operations like convolution and pooling, a multi-dimensional feature map is typically obtained. To facilitate tasks such as classification or regression, these multi-dimensional features need to be transformed into a one-dimensional vector. The architecture and information workflow of the CNN model used in this study for single-step feature extraction are illustrated in Figure 6.

Compared to other classification algorithms, CNN networks optimize kernels through automated learning, whereas in traditional algorithms, these filters are manually designed. Since every convolutional layer uses the same kernel, the network has fewer parameters, reducing model complexity while enhancing generalization capability [40].

#### 2.5.2. SVM Principle

**Figure 7:**
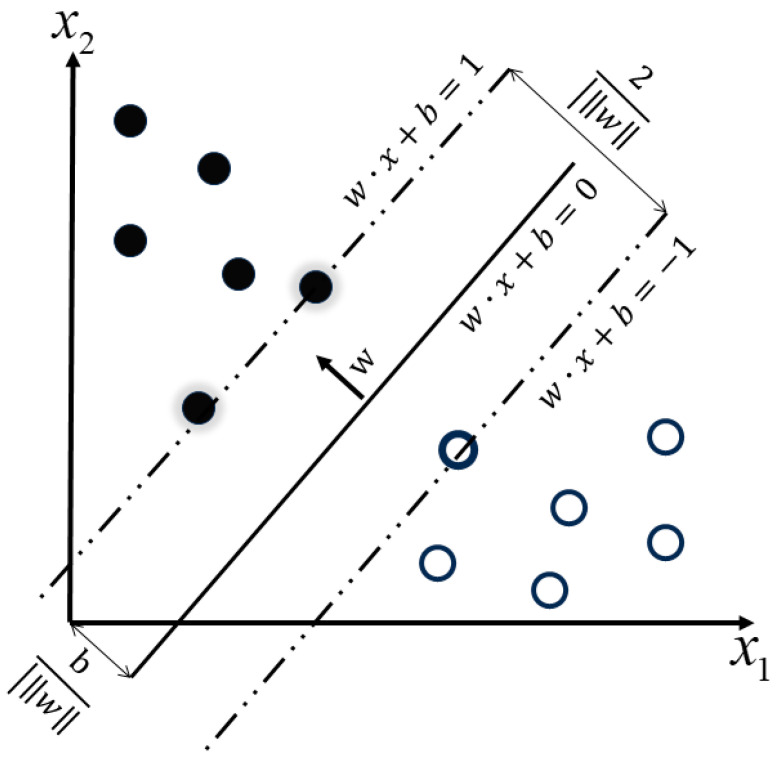
SVM hyperplane division.

The support vector machine (SVM) is a classification method pioneered by Vladimir Vapnik and his colleagues at AT&T Bell Labs [41]. Its foundational principle is to find the optimal classification hyperplane, especially for linearly separable datasets. The SVM aims to ensure the closest data points to this hyperplane from each class are maximally distant, thus maximizing the margin between classes. For non-linearly separable datasets, the SVM employs a technique called the “kernel trick”. This method non-linearly maps samples from a lower-dimensional space to a higher-dimensional one, making them linearly separable. The classification principle is depicted in Figure 7.

In Figure 7, the solid and hollow circles respectively represent two classes of samples. The hyperplane that separates them can be represented as:

(5) w·x+b=0

where x is the feature vector of the input sample, w is the normal vector of the hyperplane, and b is the intercept of the hyperplane.

Compared to the Softmax classifier, the SVM exhibits greater robustness. This is because its decision boundary is primarily determined by the support vectors, rather than all data points, allowing the SVM to perform better in the presence of noisy data and outliers.

#### 2.5.3. CNN–SVM Principle

Unlike traditional manual feature extraction methods, CNNs can automatically learn feature representations, thereby reducing the intricacies of feature engineering. While traditional approaches necessitate domain experts to select and design features manually, CNNs extract discriminative features directly from raw data through convolution and pooling, without the need for human intervention. This autonomous feature learning equips CNNs with the efficiency to manage large-scale and intricate data, ensuring adaptability across various tasks and data distributions. The weight sharing and local receptive fields inherent to CNNs also lead to a reduction in model parameters, diminishing overfitting risks and accelerating model training and inference processes. Nonetheless, a standard CNN’s Softmax classifier may underperform when faced with non-linear output challenges [42]. To mitigate this, our research incorporates an SVM as the classifier. Utilizing kernel functions, SVM can transmute non-linear classification boundaries in the primary feature space to linear counterparts in a higher-dimensional space, resulting in enhanced classification outputs.

**Figure 8:**
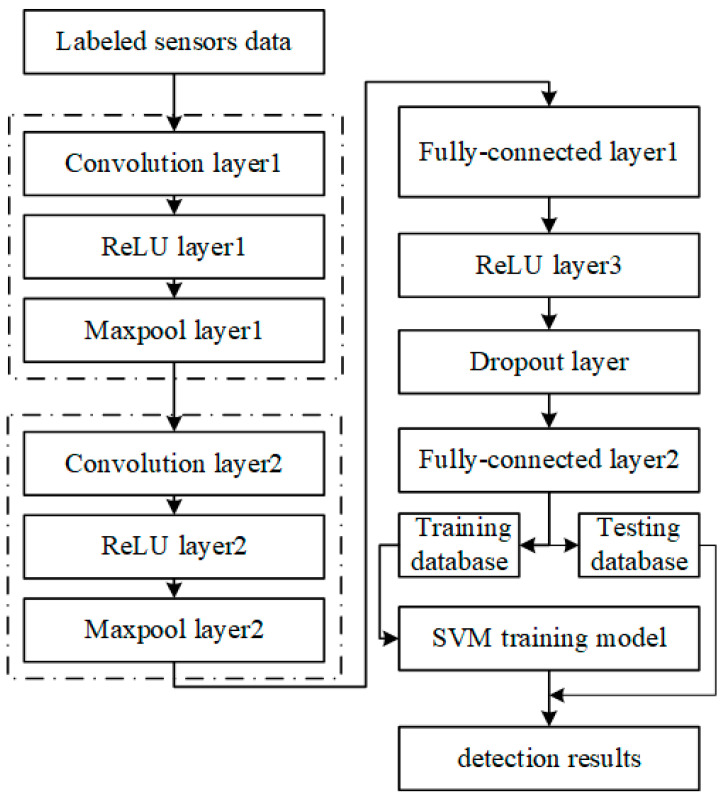
CNN–SVM network structure.

The fundamental principle of our CNN–SVM model lies in the substitution of the CNN’s fully connected layer with an SVM. Essentially, after feature extraction by CNN, the classification task is entrusted to SVM. The raw data is first processed by the CNN to procure feature vectors, which are then segmented into training and testing subsets. During the offline training phase, this training dataset is utilized to train the SVM network. In the testing phase, the testing dataset is transformed into feature vectors, which are then classified by the SVM trained model. The model’s architecture is elucidated in Figure 8.

### 2.6. Heading Angle Fusion

In the indoor PDR algorithm’s heading estimation phase, challenges emerge, notably from magnetic interference impacting the magnetometer and biases present in gyroscope measurements. Traditional smartphone system application interfaces (APIs) have shown that the heading estimation error can escalate to around 20 degrees in a span of mere minutes [43]. This section, anchored on the classification outcomes of the CNN–SVM model, introduces varied fusion methods to amalgamate the headings derived from the gyroscope and the magnetometer. Specifically, in the absence of magnetic interference, the UKF method merges the magnetometer and gyroscope readings. However, when magnetic interference is present, fusion between the previous heading and the current gyroscope heading is carried out to minimize the impact of the magnetic interference, serving as a countermeasure against this interference.

#### 2.6.1. Heading Estimation without Magnetic Interference

Pedestrian motion is inherently random, which presents challenges when using the Euler method to calculate attitude angles due to potential gimbal lock issues. Additionally, the intrinsic non-linearity of the quaternion motion model complicates attitude estimation [44]. An effective approach to address these challenges is the direction cosine matrix, which converts the dynamic rigid body coordinate system transformation into matrix operations. This method enhances the precision of attitude estimation and control. In this paper, we adopt the TRIAD algorithm [45], utilizing data from a smartphone’s magnetometer and accelerometer to derive the attitude. The underlying principle of this algorithm is delineated as follows:

(6) $$A_{n}=\left[\begin{array}{ccc} g^{n} & \frac{g^{n}\times m^{n}}{\left\|g^{n}\times m^{n}\right\|} & \frac{g^{n}\times (g^{n}\times m^{n})}{\left\|g^{n}\times (g^{n}\times m^{n})\right\|} \end{array}\right]$$

(7) $$A_{b}=\left[\begin{array}{ccc} a^{b} & \frac{a^{b}\times m^{b}}{\left\|a^{b}\times m^{b}\right\|} & \frac{a^{b}\times (a^{b}\times m^{b})}{\left\|a^{b}\times (a^{b}\times m^{b})\right\|} \end{array}\right]$$

In the formula, $g^{n}=\left[\begin{array}{ccc} 0 & 0 & -1 \end{array}\right]$; $m^{n}=\left[\begin{array}{ccc} m_{x} & 0 & m_{z} \end{array}\right]$; $m_{x}{}^{2}{+m}_{z}{}^{2}=1$. $a^{b}$ represents the accelerometer measurement data; and $m^{b}$ represents the magnetometer measurement data. $m^{b}$ and $g^{n}$ are projections in the Earth’s navigation coordinate system. Additionally, $A_{n}=RA_{b}$, $g^{n}=Ra^{b}$, and $m^{n}=Rm^{b}$. Ultimately, the relationship between the attitude rotation matrix and the matrix constituted by the measurement vector is defined as:

(8) $$R=A_{n}A_{b}{}^{-1}$$

From the properties of the attitude rotation matrix, we know that $R^{-1}=R^{\prime }$, leading to $R=(A_{b}A_{n}{)}^{\prime }=\left[\begin{array}{ccc} x_{11} & x_{12} & x_{13}\\ x_{21} & x_{22} & x_{23}\\ x_{31} & x_{32} & x_{33} \end{array}\right]$. From R, we can derive that during a pedestrian’s movement, the Euler angles obtained from the smartphone are:

(9) $$\mathrm{Pitch}\theta =a\sin(-x_{33})$$

(10) $$\mathrm{Roll}\phi =a\tan2\left(x_{32},\;x_{31}\right)$$

(11) $$\mathrm{Yaw}\psi =a\tan2\left(x_{13},\;x_{23}\right)$$

In this study, we incorporated angular velocity from the gyroscope to determine the roll, pitch, and yaw angles. The equations are as follows:

(12) $$\mathrm{Pitch}\theta_{k+1}^{g}=\theta_{k}^{g}+\int p_{k}^{}d\Delta t$$

(13) $$\mathrm{Roll}\phi_{k+1}^{g}=\phi_{k}^{g}+\int q_{k}^{}d\Delta t$$

(14) $$\mathrm{Yaw}\psi_{k+1}^{g}=\psi_{k}^{g}+\int r_{k}^{}d\Delta t$$

where $\theta_{k+1}^{g}$, $\phi_{k+1}^{g}$ and $\psi_{k+1}^{g}$ represents the pitch, roll, and yaw angle of the gyroscope at time k + 1. $p_{k}^{}$, $q_{k}^{}$, and $r_{k}^{}$ denote the x-, y-, and z-axis accelerations of the gyroscope at time k, respectively. Δt is the sampling time interval.

It is well-understood that gyroscopes, over extended periods, exhibit drift issues. Concurrently, magnetometers (or compasses) are prone to inaccuracies in magnetically noisy environments, resulting in unstable heading angle estimates. To address these challenges, we utilize the UKF to integrate data from both the gyroscope and magnetometer. This fusion approach capitalizes on the individual strengths of each sensor while compensating for their inherent weaknesses, enabling us to derive more robust and accurate heading angle estimates [46].

#### 2.6.2. Heading Estimation under Magnetic Interference

In the presence of magnetic interference, the gyroscope offers a short-term, accurate heading estimation. In this context, our approach considers the unaffected heading from the previous step. This heading is then adjusted by adding the change recorded by the gyroscope post-interference to yield the final heading for the current step. The underlying mathematical model is detailed below:

(15) $$\psi_{k}=\psi_{k-1}+\Delta \psi_{k}^{g}$$

In the formula, $\psi_{k}$ represents the final heading at step k, and $\Delta \psi_{k}^{g}=\psi_{k}^{g}-\psi_{k-1}^{g}$ denotes the change in gyroscope heading from step k − 1 to step k.

### 2.7. Accuracy Evaluation Metrics

In this article, the absolute error of the pedestrian’s single-step heading angle is used to measure the accuracy of the model’s heading angle calculation. Its formula is:

(16) $$E_{h}=abs(H_{e}^{i}-H_{t}^{i})$$

In the formula, $H_{e}^{i}$ and $H_{t}^{i}$ represent the calculated heading value and the true heading value for the ith step.

To evaluate the error in the positioning results, this study employs three metrics: average distance error, walking distance error rate, and Fréchet distance. Their respective formulas are as follows:

(17) $$\mathrm{Average}\;\mathrm{distance}\;\mathrm{error}:\;AE=\frac{1}{N}\sum_{i=1}^{N}\left(\sqrt{{\left(x_{e}^{i}-x_{t}^{i}\right)}^{2}+{\left(y_{e}^{i}-y_{t}^{i}\right)}^{2}}\right)$$

(18) $$\mathrm{Walking}\;\mathrm{distance}\;\mathrm{error}\;\mathrm{rate}:\;RAE=\frac{1}{N}\sum_{i=1}^{N}\left(\sqrt{{\left(x_{e}^{i}-x_{t}^{i}\right)}^{2}+{\left(y_{e}^{i}-y_{t}^{i}\right)}^{2}}\right)/L$$

(19) $$\mathrm{Fréchet}\;\mathrm{distance}:\;d_{F}(P,Q)=\min_{\alpha ,\beta }\max_{i\in [1,n],j\in [1,m]}\left\|P(i)-Q(j)\right\|$$

In the formula, $x_{e}^{i}$ represents the estimated eastward coordinate of the ith step; $x_{t}^{i}$ represents the actual eastward coordinate of the ith step; $y_{e}^{i}$ stands for the estimated northward coordinate of the ith step; $y_{t}^{i}$ represents the actual northward coordinate of the ith step; N is the total number of steps; and L represents the total walking distance. Let P and Q denote two curves, where P consists of n points and Q consists of m points. α:[1,n]→[1,n] and β:[1,m]→[1,m] are non-decreasing mappings that satisfy α(1)=1,α(n)=n,β(1)=1 and β(n)=n.

## 3. Results

### 3.1. Comparative Data Analysis

**Figure 9:**
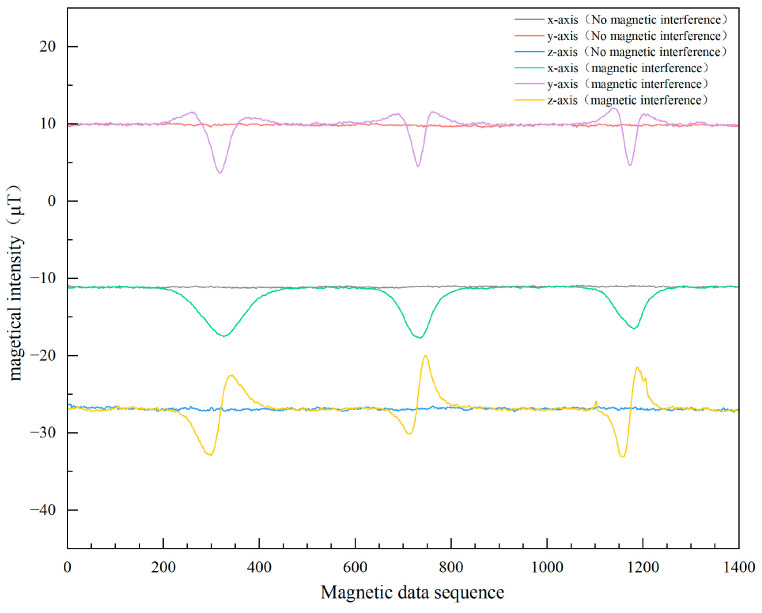
Comparison chart of magnetometer three-axis component data with and without magnetic interference in a stationary environment.

To ascertain the influence of the iron key on the smartphone’s magnetometer, the study followed a two-phase approach. First, the smartphone was kept stationary, and the iron key was moved back and forth close to it, recording magnetometer data across all three axes under this interference. Secondly, data was collected from the smartphone’s magnetometer in the absence of the iron key to serve as a baseline. A comparative analysis of these data sets is presented in Figure 9, showcasing the extent of interference caused by the iron key.

**Figure 10:**
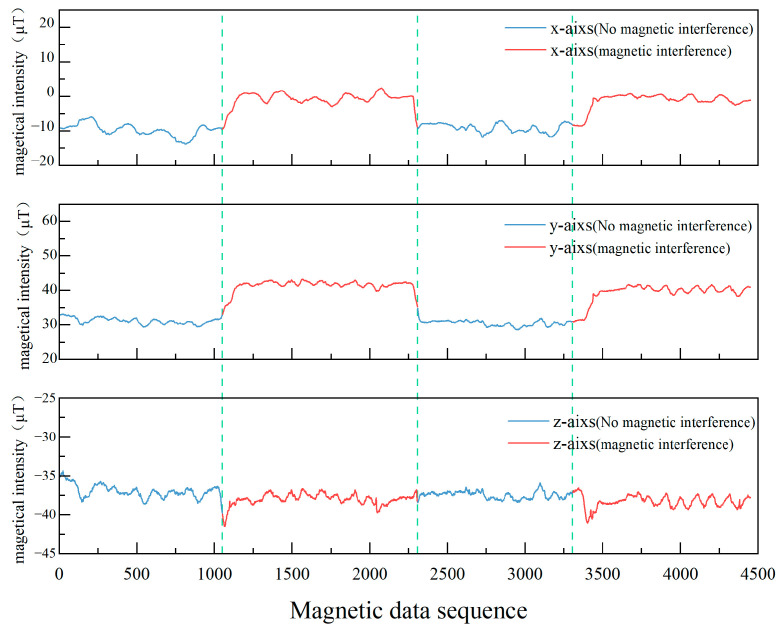
Comparison of the magnetometer’s behavior on a straight path with and without magnetic interference.

We know that when a pedestrian holds a smartphone with a constant posture and walks along a straight path, the data from the three axes of the magnetometer will form a straight line. However, changes in the local magnetic field can lead to fluctuations in the magnetometer data. The results are shown in Figure 10.

From Figure 9 and Figure 10, it is evident that the magnetometer in smartphones is susceptible to interference from iron objects, causing significant disruptions in its normally stable data. Such disruptions can compromise the efficacy of the PDR’s magnetometer heading. To bolster pedestrian positioning accuracy, it is crucial to detect magnetic interference and adeptly integrate the heading angle.

**Figure 11:**
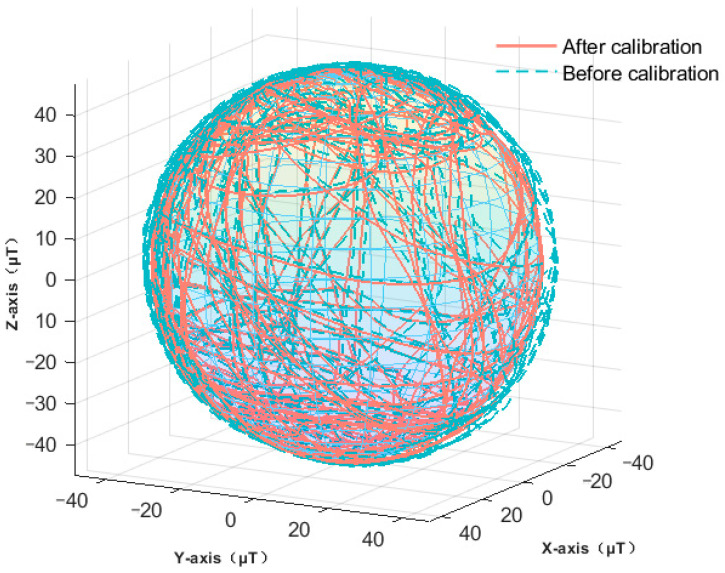
Comparison of magnetometer data before and after calibration.

Figure 11 illustrates the contrast between the magnetometer data pre- and post-calibration in this investigation. Through this calibration process, both hard-iron and soft-iron distortions intrinsic to the magnetometer are rectified. Consequently, the offset and scaling inaccuracies have been addressed. As depicted in the figure, post-calibration data better conforms to the expected geomagnetic field pattern. This enhancement bolsters the magnetometer’s precision, ensuring its capability to reliably discern magnetic field nuances in real-world scenarios. By mitigating error impacts, we achieve a more precise heading determination.

### 3.2. Model Accuracy Comparison

**Figure 12:**
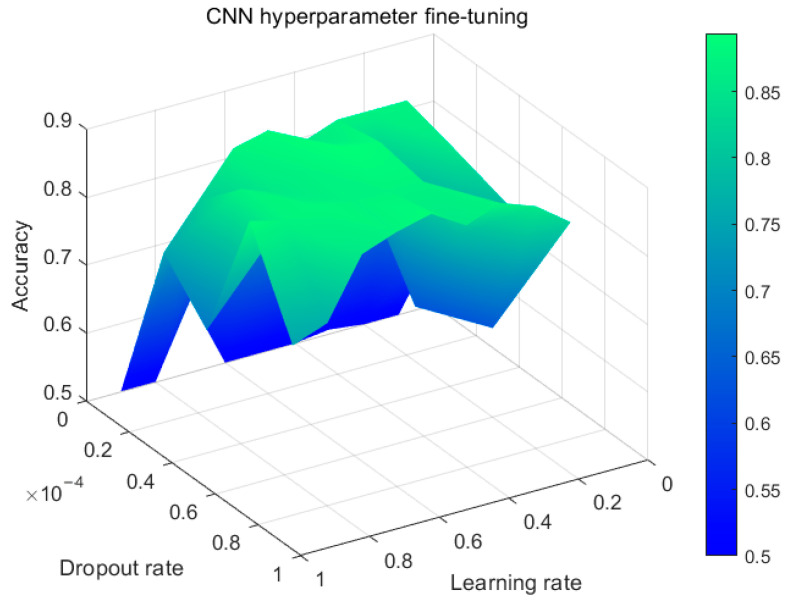
Hyperparameter fine-tuning for the CNN.

For the CNN model section, this study employs the ”sgdm” optimizer with 100 iterations. Based on the grid search results shown in Figure 12, the learning rate is set to 0.4 × 10^−4^ and the dropout rate is set to 0.5.

**Figure 13:**
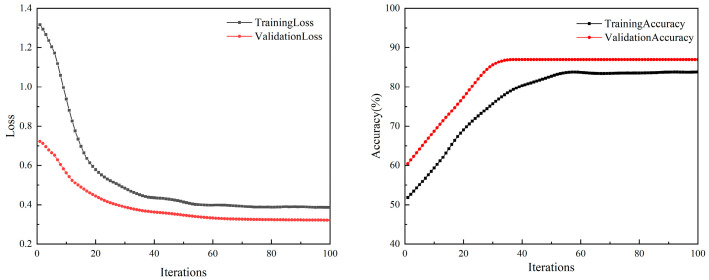
Comparison of training loss, validation loss, training accuracy, and validation accuracy for the CNN model.

The architecture of the CNN comprises two main layers. The first convolutional layer consists of 32 kernels, each with a size of 3 and a stride of 1 × 1, paired with ReLU as the activation function. Following this layer is a max-pooling layer, characterized by a 2 × 2 pooling window and a 2 × 2 stride. Subsequently, the second convolutional layer features 64 kernels, maintaining a kernel size of 3 and a stride of 1 × 1, with ReLU activation once again. This layer is followed by another max-pooling layer with similar parameters to the first: a 2 × 2 pooling window and a 2 × 2 stride. The fully connected layer transforms the features into a one-dimensional vector, followed by a dropout layer to prevent overfitting. The training loss and accuracy curves are shown in Figure 13.

As can be seen from Figure 13, both the training loss and validation loss show a decreasing trend, while the training accuracy and validation accuracy are on the rise. During the training process, the validation accuracy tends to stabilize after 45 iterations, with no evident signs of overfitting.

**Figure 14:**
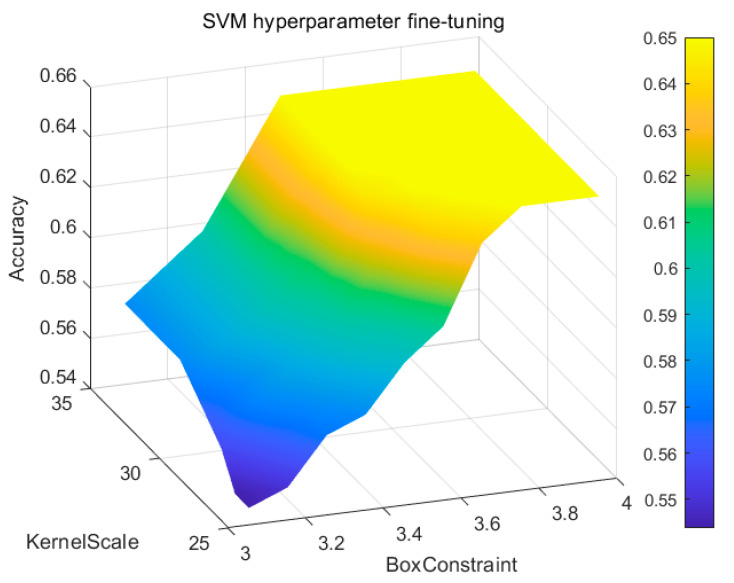
Hyperparameter tuning for the SVM.

In the SVM portion of our model, the RBF (radial basis function) Gaussian kernel was selected. Based on the cross-validation results shown in Figure 14 using grid search, the penalty coefficient C was determined to be 3.4719, and the kernel coefficient gamma was set to 29.5068.

This integrated CNN–SVM model, harnessing the strengths of both CNN and SVM frameworks, demonstrated a remarkable accuracy of 99.38%. For a comparative analysis of the performance metrics, including accuracy and detection time across three distinct network architectures, please refer to Table 1.

From Table 1, it is evident that the CNN–SVM model proposed in this study achieves a detection accuracy of 99.38% for the presence of local magnetic field interference, with an F1 score of 99.37%. This performance is notably superior to that of standalone CNN or SVM models. Concurrently, while the training time for the CNN–SVM model is noticeably longer than that of the CNN model alone, it is approximately the sum of the training times for both the CNN and SVM models, clocking in at 13.5250 s. This is attributed to the integration of both CNN and SVM algorithms. However, once the model is established, the CNN–SVM model can swiftly and accurately detect local magnetic field interference, lagging only 0.0214 s behind the fastest SVM model and significantly outperforming the detection time of the CNN model.

### 3.3. Comparison of Heading Estimation Accuracy

To evaluate the performance of the proposed integrated heading estimation algorithm, walking route experiments were conducted indoors. The magnetometer heading, gyroscope heading, and optimal UKF heading estimation method from the literature [21] were compared.

**Figure 15:**
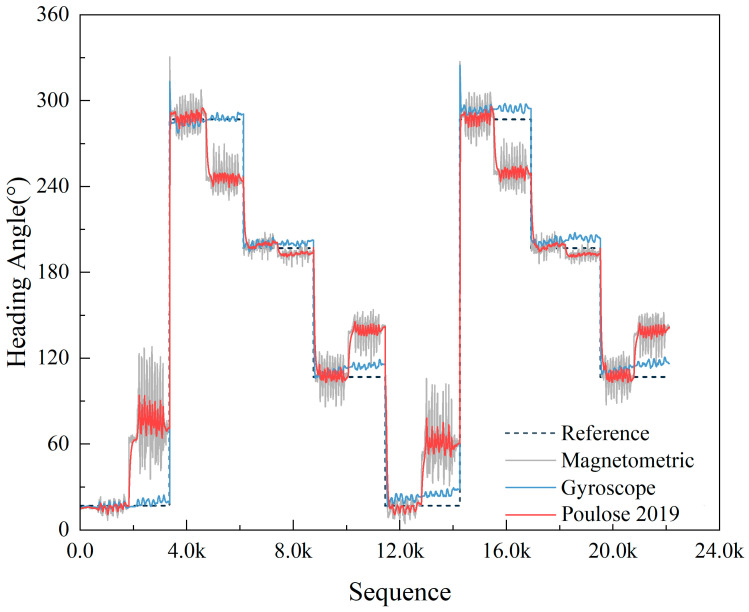
Comparison of heading angles from UKF fusion [21], standalone magnetometer, and standalone gyroscope data.

In scenarios without magnetic interference, we use the UKF method proposed in reference [21] to fuse the heading angles from the gyroscope and magnetometer. The heading results are based on data collected during two rounds of motion on a pre-established trajectory, as shown in Figure 15.

The gyroscope, being unable to determine the absolute heading angle, can only measure the change in angle during walking. In this study, the initial heading angle obtained from the magnetometer data was used as the initial value for the gyroscope heading. As evident from Figure 15, the gyroscope remains largely unaffected by magnetic interference, though it demonstrates some heading drift. On the other hand, magnetometers suffer from substantial noise and are significantly impacted by magnetic disturbances. Particularly when under magnetic interference, the heading information from the magnetometer becomes almost unusable. In the absence of magnetic interference, the UKF algorithm employed in this paper can to some extent mitigate the severe noise in the magnetometer heading and correct the gyroscopic heading drift. However, under magnetic interference, due to the unreliability of the magnetometer’s heading, the fused result deviates from the actual path.

**Figure 16:**
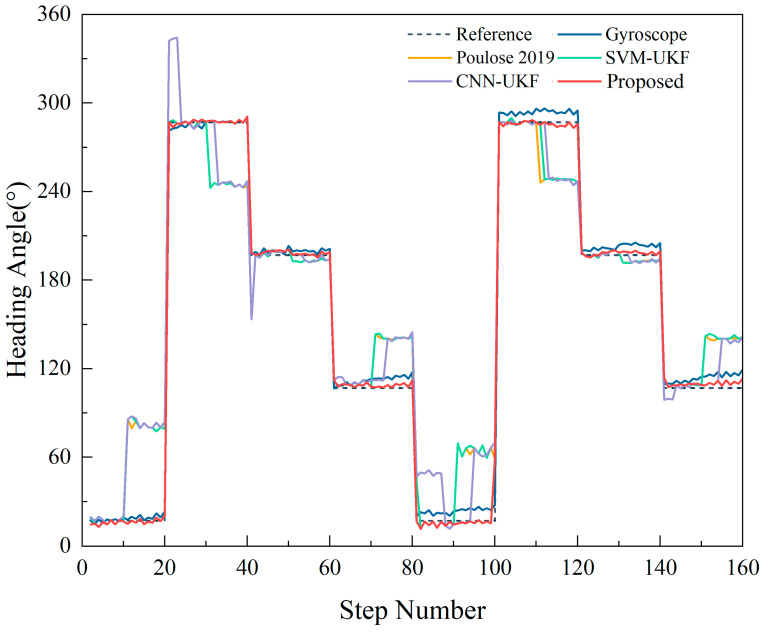
Comparison of the heading angles obtained from three detection methods: CNN–SVM, CNN, and SVM [21].

Consequently, based on step frequency detection, this paper adopts the average heading within a single step as the heading for that step. Combining the magnetic interference detection results from the CNN, SVM, and CNN–SVM models, in situations without magnetic interference, the UKF-fused heading is used. Conversely, during magnetic interference, the heading angle is calculated using Equation (15), resulting in the heading angle as shown in Figure 16.

Figure 16 clearly shows the effectiveness of the proposed CNN–SVM–UKF heading estimation in this study. It not only effectively combats the inaccuracy of the UKF-fused heading due to magnetic interference in the reference [21] but also addresses the initial value requirement of the gyroscope heading and significantly minimizes its drift. Using these refined heading estimates, we determined the absolute heading error per step for different acquisition settings, as shown in Table 2.

Table 2 reveals that in the absence of magnetic interference, the UKF-fused heading angle’s absolute error is a mere 1.7948°. This represents a 51.35% and 53.77% accuracy improvement over gyroscopic and magnetometer headings, respectively. While single models like CNN–UKF and SVM–UKF falter under magnetic interference, the CNN–SVM–UKF model shines with a single-step absolute heading error of just 2.1891°—an impressive 61.67% accuracy improvement compared to the unaffected gyroscopic heading. Such progress underscores the enhanced resistance to magnetic interference in smartphone heading estimation, leading to substantially more accurate results.

### 3.4. Pedestrian Motion Trajectory Estimation

**Figure 17:**
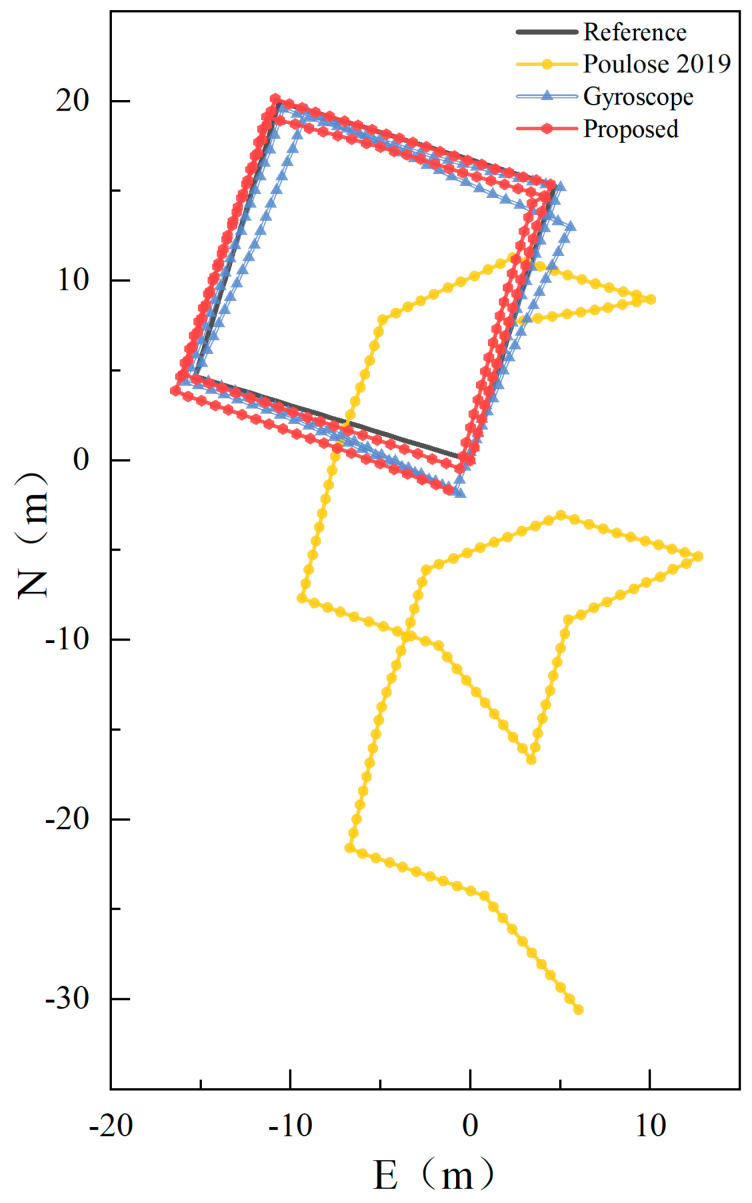
Localization results of square trajectory in a local magnetic field interference environment [21].

In this section, following the principles of PDR, when the pedestrian’s movement is affected by local magnetic field interference, the pedestrian’s motion trajectory, as shown in Figure 17, is obtained based on the heading estimated by the CNN–SVM–UKF model proposed in this study and the Weinberg step length calculated from the acceleration data.

**Figure 18:**
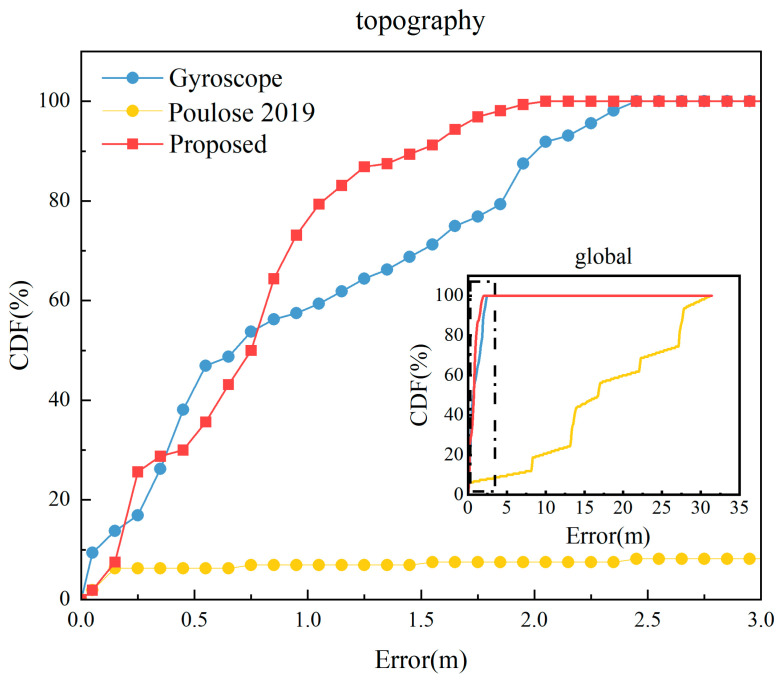
Cumulative distribution of localization errors for our algorithm, traditional gyroscope, and UKF heading [21]. (Zoomed-in view of the black dot line within the global image).

From Figure 17, it is evident that even with interference in the smartphone’s local magnetic field, the methodology presented in this paper closely aligns with the pedestrian’s movement trajectory. Compared to the gyroscope heading localization results, which are unaffected by local magnetic field interference, our approach not only mitigates the gyroscope heading drift but also further reduces the localization error. In contrast, the fusion heading localization results from the literature [21] significantly deviate from the actual pedestrian movement trajectory, making it challenging to meet the indoor pedestrian localization requirements when subjected to local magnetic field disturbances. This underscores the effectiveness of our CNN–SVM–UKF method in detecting local magnetic field interference, thereby enhancing indoor pedestrian localization accuracy. The CDF comparison of its localization error is depicted in Figure 18.

From Figure 18, it can be observed that the maximum localization error of our proposed algorithm is 2.0402 m, which is significantly less than the 31.1481 m error exhibited by the method from the literature [21] under local magnetic field interference. It also outperforms the maximum error of 2.4761 m from the gyroscope heading unaffected by local magnetic field interference, reducing the maximum error by 17.60%. Under conditions where the local magnetic field is disturbed, 80% of the localization errors obtained using our algorithm are less than 1.1000 m, whereas for the gyroscope heading, 80% of the errors are less than 1.9232 m, marking a reduction of 42.80%.

Through the analysis of error data from different algorithms on the square path, we obtained the results shown in Table 3.

From Table 3, it is evident that under the influence of local magnetic field disturbances, the average positioning error of our proposed algorithm is a mere 0.7865 m. This represents a 20.6997% enhancement in accuracy compared to the gyroscope-based heading and a significant 95.4442% improvement over the UKF heading method outlined in the literature [21]. In terms of walking distance error rate, our method demonstrates a 0.16% reduction compared to the gyroscope heading, which translates to an improved distance estimation accuracy of 0.16 m for every meter traveled. Furthermore, our algorithm exhibits the highest trajectory similarity when juxtaposed with the other two methods, marking a 32.72% improvement over the gyroscope heading’s trajectory resemblance.

**Figure 19:**
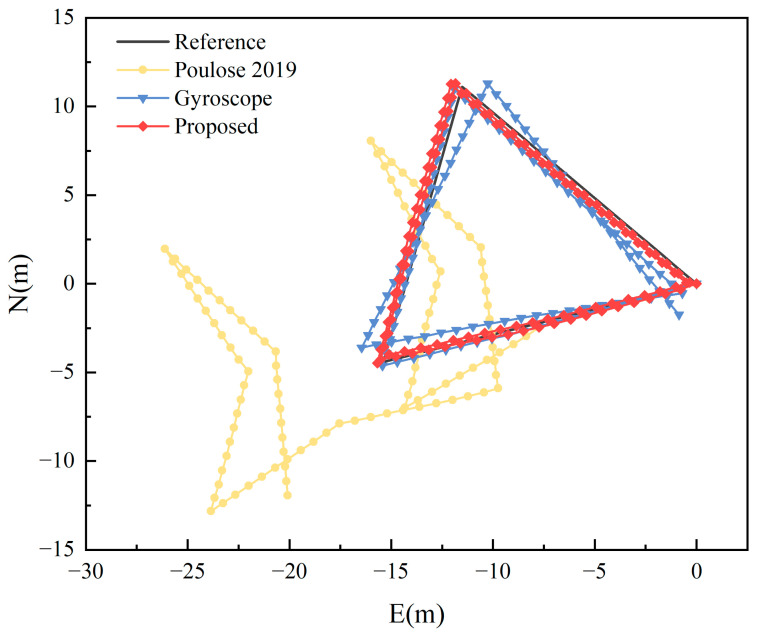
Positioning results for the equilateral triangle path under local magnetic field interference [21].

**Figure 20:**
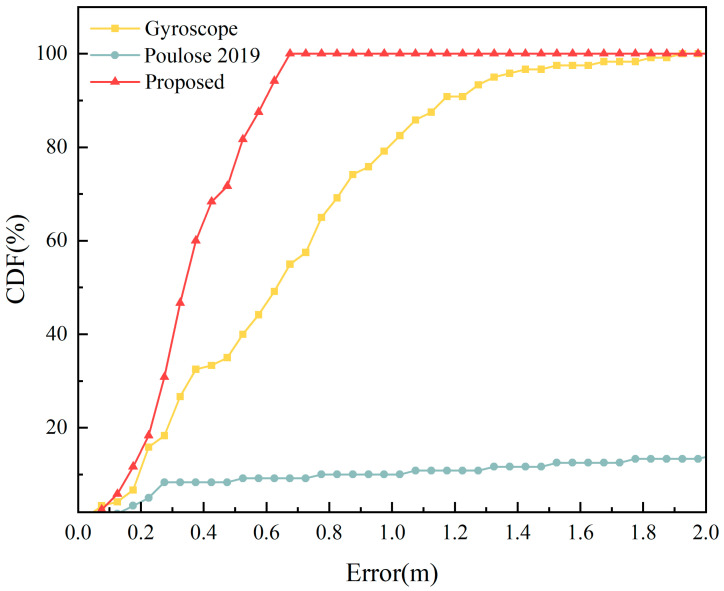
CDF of positioning errors for the equilateral triangle path under local magnetic field interference [21].

To delve deeper into the adaptability of our method across different paths, we expanded our data collection approach, as described in Section 2.3, to encompass an equilateral triangle path with a side length of 16 m. A comparative analysis of the trajectory and cumulative error between our method, the gyroscope heading, and the approach from the literature [21] under local magnetic field interference is depicted in Figure 19 and Figure 20.

From Figure 19, it is evident that the proposed algorithm in this study effectively mitigates the impact of local magnetic interference on indoor pedestrian positioning, aligning more closely with the actual trajectory of the pedestrian. The mean absolute error for the single-step heading is 1.5805°. As illustrated in Figure 20, the maximum positioning error of our method is 0.6847 m. In comparison to the gyroscope heading’s maximum error of 1.9492 m, there is an accuracy improvement of 64.87%. For our method, 80% of the positioning errors are less than 0.4377 m, whereas for the gyroscope heading, 80% of the errors are less than 0.8316 m. In the context of local magnetic interference, only 10% of the errors in the literature [21] are less than 0.8 m, making the method unsuitable for indoor pedestrian positioning. A comparison of positioning errors based on the three heading estimation methods is presented in Table 4.

From Table 4, it is evident that under the influence of local magnetic interference, the algorithm proposed in this study outperforms both the method from the literature [21] and the gyroscope heading-based positioning in terms of average positioning error, average walking distance error, and Fréchet distance. Compared to the gyroscope heading positioning results, there is a reduction of 43.42%, 43.41%, and 31.57% respectively.

Considering the results from the two path experiments, it is clear that our CNN–SVM–UKF method addresses challenges such as obtaining the initial heading angle solely from the gyroscope and the influence of local magnetic interference on the magnetometer. Furthermore, it enhances the positioning accuracy of smartphones in indoor environments affected by local magnetic interference.

## 4. Discussion and Conclusions

This study leverages the MEMS sensors embedded in smartphones, including accelerometers, gyroscopes, and magnetometers, to deduce the indoor movement trajectory of pedestrians. To effectively mitigate the negative impacts of local magnetic disturbances on heading estimation, we integrated a CNN–SVM deep-learning approach for detecting such interferences. Our findings underscore that the CNN–SVM model, when juxtaposed with individual SVM or CNN networks, showcases heightened accuracy and efficiency in pinpointing local magnetic disturbances. In the realm of heading estimation, based on the CNN–SVM’s detection outcomes, we delved into the performance metrics of the heading estimation algorithm in scenarios with and without magnetic interference. Moreover, by amalgamating heading estimation with the Weinberg step-length estimation, we successfully charted the precise indoor movement path of pedestrians. Experimental validations on both square and equilateral triangular paths revealed our method’s average positioning errors to be 0.7565 m and 0.3856 m, respectively, marking a notable improvement in indoor positioning precision amid local magnetic disturbances. The salient conclusions of our research are:

(1) Model Excellence and Prospective Avenues: The CNN–SVM framework proposed herein stands out in its efficacy for detecting local magnetic disturbances, especially when contrasted with conventional standalone CNN or SVM models. This composite approach adeptly harnesses the strengths of both models, adeptly navigating the challenges posed by the CNN’s Softmax classifier in addressing intricate non-linear challenges and the SVM’s vulnerability to data redundancies. This synergy significantly bolsters the model’s adaptability and robustness. While our current methodology leans on grid search for optimal hyperparameter determination, future endeavors will explore sophisticated optimization techniques, such as genetic algorithms or particle swarm optimization, to refine model performance and adaptability.

(2) Real-time Capabilities and Application Horizons: Our CNN–SVM amalgamation shines in its real-time detection prowess. For a dataset encompassing 160 steps of local magnetic interference, the model delivers accurate detection in a mere 0.058 s, aligning with the stringent demands of real-time positioning applications. Given today’s smart devices’ computational prowess, our model holds promising application potential. Future iterations will focus on refining this detection paradigm and embedding it within smart devices, realizing genuine end-to-end, real-time magnetic interference detection and precision positioning.

(3) Experimental Environment Selection: We simulated a common indoor positioning scenario where the local magnetic field is disturbed by keys held in the hand while a person is walking. To eliminate uncertain factors in the surrounding environment, we chose an idealized setting with minimal magnetic interference from iron doors, windows, or electronic devices. However, we must acknowledge that this approach has certain limitations. Specifically, this study only considered the impact of keys, which are iron objects, on the estimation of a smartphone’s heading. In real-world applications, the scenario is not so idealized, and the magnetic field around a person could be affected by iron doors, windows, or electronic devices, causing changes. Nevertheless, this research still provides an important perspective for the estimation of smartphone navigation and indoor positioning in environments where the local magnetic field is disturbed. In the next steps, we will consider the diversity of scenarios and the unknown effects of different magnetic interference sources.

(4) Experimental Constraints and Forward-looking Research Directions: Our experimental setup necessitated participants to consistently position the smartphone in front of their chests. This specific holding technique might not encapsulate the myriad ways pedestrians might handle their devices in real-life scenarios, introducing certain constraints. To more comprehensively consider real-world application scenarios, we plan to further investigate the performance of heading estimation for smartphones in various carrying postures and for pedestrians in different activity modes. We will also consider the impact of multiple interference sources on the magnetic field, continuously enhancing the versatility of our model. This will aid us in further optimizing and improving the positioning accuracy for pedestrians in a variety of real-world situations.

## Figures and Tables

**Table 1:** Comparison of Magnetic Interference Detection Accuracy Across Three Networks.

Model	CNN	SVM	CNN–SVM
Accuracy Rate (%)	89.38%	65.00%	99.38%
F1 (%)	88.89%	60.42%	99.37%
Offline Training Time (s)	13.139411	0.416145	13.5250
Online Detection Time (s)	0.603868	0.036875	0.058248

**Table 2:** Comparison Table of Single-Step Heading Absolute Errors.

Attribute	Absence of Magnetic Interference			Under Magnetic Interference			
	Gyroscope	Magnetometer	UKF	Gyroscope	SVM–UKF	CNN–UKF	Proposed
Average (°)	3.6895	3.8822	1.7948	5.7115	33.4564	26.5877	2.1891

**Table 3:** Comparison table of errors for different algorithms on the square path.

Model	Literature [21]	Gyroscope	Proposed
AE(m)	17.2639	0.9918	0.7865
RAE(%)	13.4874%	0.7748%	0.6145%
$d_{F}(P,Q)$ (m)	230.9076	13.2120	8.8995

**Table 4:** Comparison table of errors for different algorithms on the equilateral triangle path.

Model	Literature [21]	Gyroscope	Proposed
AE (m)	9.2753	0.6815	0.3856
RAE (%)	9.6618%	0.7099%	0.4017%
$d_{F}(P,Q)$ (m)	101.1502	6.4057	4.3837

## Data Availability

Data are contained within the article.

## References

[1] Harle R. (2013). A Survey of Indoor Inertial Positioning Systems for Pedestrians. IEEE Commun. Surv. Tutor..

[2] Sadhukhan P., Dahal K., Das P.K. (2022). A Novel Weighted Fusion based Efficient Clustering for Improved Wi-Fi Fingerprint Indoor Positioning. IEEE Trans. Wirel. Commun..

[3] Szyc K., Nikodem M., Zdunek M. (2023). Bluetooth low energy indoor localization for large industrial areas and limited infrastructure. Ad. Hoc. Netw..

[4] Fontaine J., Van Herbruggen B., Shahid A., Kram S., Stahlke M., De Poorter E. (2023). Ultra Wideband (UWB) localization using active CIR-based fingerprinting. IEEE Commun. Lett..

[5] Xu S., Wu Y., Wang X., Wei F. (2023). Indoor High Precision Positioning System Based on Visible Light Communication and Location Fingerprinting. J. Lightwave Technol..

[6] Xiao C., Yang D., Chen Z., Tan G. (2017). 3-D BLE Indoor Localization Based on Denoising Autoencoder. IEEE Access.

[7] Sun M., Wang Y., Huang L., Jia H., Bi J., Joseph W., Plets D. (2022). Geomagnetic positioning-aided Wi-Fi FTM localization algorithm for NLOS environments. IEEE Commun. Lett..

[8] Zheng H., Gao M., Chen Z., Liu X., Feng X. (2019). An adaptive sampling scheme via approximate volume sampling for fingerprint-based indoor localization. IEEE Internet Things J..

[9] Hou X., Bergmann J. (2020). Pedestrian dead reckoning with wearable sensors: A systematic review. IEEE Sens. J..

[10] Bao S., Meng X., Xiao W., Zhang Z. (2017). Fusion of inertial/magnetic sensor measurements and map information for pedestrian tracking. Sensors.

[11] Wu Y., Zhu H., Du Q., Tang S. (2018). A pedestrian dead-reckoning system for walking and marking time mixed movement using an SHSs scheme and a foot-mounted IMU. IEEE Sens. J..

[12] Xie L., Tian J., Ding G., Zhao Q. (2017). Holding-manner-free heading change estimation for smartphone-based indoor positioning. Proceedings of the 2017 IEEE 86th Vehicular Technology Conference (VTC-Fall).

[13] Zhou R. (2016). Pedestrian dead reckoning on smartphones with varying walking speed. Proceedings of the 2016 IEEE International Conference on Communications (ICC).

[14] Afzal M.H., Renaudin V., Lachapelle G. (2011). Magnetic field based heading estimation for pedestrian navigation environments. Proceedings of the 2011 International Conference on Indoor Positioning and Indoor Navigation.

[15] Alatise M.B., Hancke G.P. (2017). Pose estimation of a mobile robot based on fusion of IMU data and vision data using an extended Kalman filter. Sensors.

[16] Bravo J., Herrera E.P., Sierra D.A. (2017). Comparison of step length and heading estimation methods for indoor environments. Proceedings of the 2017 IEEE XXIV International Conference on Electronics, Electrical Engineering and Computing (INTERCON).

[17] Nguyen P., Akiyama T., Ohashi H., Nakahara G., Yamasaki K., Hikaru S. (2016). User-friendly heading estimation for arbitrary smartphone orientations. Proceedings of the 2016 International Conference on Indoor Positioning and Indoor Navigation (IPIN).

[18] Yang X., Huang B., Miao Q. (2016). A step-wise algorithm for heading estimation via a smartphone. Proceedings of the 2016 Chinese Control and Decision Conference (CCDC).

[19] Ma W., Wu J., Long C., Zhu Y. (2015). HiHeading: Smartphone-based indoor map construction system with high accuracy heading inference. Proceedings of the 2015 11th International Conference on Mobile Ad-hoc and Sensor Networks (MSN).

[20] Wu Y., Zou D., Liu P., Yu W. (2017). Dynamic magnetometer calibration and alignment to inertial sensors by Kalman filtering. IEEE Trans. Control. Syst. Technol..

[21] Poulose A., Senouci B., Han D.S. (2019). Performance Analysis of Sensor Fusion Techniques for Heading Estimation Using Smartphone Sensors. IEEE Sens. J..

[22] El-Diasty M. (2014). An accurate heading solution using MEMS-based gyroscope and magnetometer integrated system (preliminary results). Ann. Photogramm. Remote Sens. Spat. Inf. Sci..

[23] Farahan S.B., Machado J.J.M., de Almeida F.G., Tavares J.M.R.S. (2022). 9-DOF IMU-Based Attitude and Heading Estimation Using an Extended Kalman Filter with Bias Consideration. Sensors.

[24] Shin E., El-Sheimy N. (2004). An unscented Kalman filter for in-motion alignment of low-cost IMUs. Proceedings of the PLANS 2004. Position Location and Navigation Symposium (IEEE Cat. No. 04CH37556).

[25] Tian J., Cong L., Qin H. (2022). A PDR heading estimation method based on motion mode recognition using adaptive UKF. Proceedings of the 2022 IEEE 12th International Conference on Indoor Positioning and Indoor Navigation (IPIN).

[26] Pei L., Liu D., Zou D., Choy R.L.F., Chen Y., He Z. (2018). Optimal heading estimation based multidimensional particle filter for pedestrian indoor positioning. IEEE Access.

[27] Valenti R.G., Dryanovski I., Xiao J. (2015). Keeping a good attitude: A quaternion-based orientation filter for IMUs and MARGs. Sensors.

[28] Robert-Lachaine X., Mecheri H., Larue C., Plamondon A. (2017). Effect of local magnetic field disturbances on inertial measurement units accuracy. Appl. Ergon..

[29] Suh Y.S., Ro Y.S., Kang H.J. (2012). Quaternion-based indirect Kalman filter discarding pitch and roll information contained in magnetic sensors. IEEE Trans. Instrum. Meas..

[30] Vandermeeren S., Steendam H. (2022). Deep-Learning-Based Step Detection and Step Length Estimation with a Handheld IMU. IEEE Sens. J..

[31] Tu P., Li J., Wang H., Wang K., Yuan Y. (2021). Epidemic contact tracing with campus WiFi network and smartphone-based pedestrian dead reckoning. IEEE Sens. J..

[32] Sangenis E., Jao C., Shkel A.M. (2022). SVM-based Motion Classification Using Foot-mounted IMU for ZUPT-aided INS. Proceedings of the 2022 IEEE Sensors.

[33] Yao Y., Liu Y., Zhou Z., Xu X. (2023). A Magnetic Interference Detection-Based Fusion Heading Estimation Method for Pedestrian Dead Reckoning Positioning. IEEE Sens. J..

[34] Yang Y., Huang B., Xu Z., Yang R. (2023). A Fuzzy Logic-Based Energy-Adaptive Localization Scheme by Fusing WiFi and PDR. Wirel. Commun. Mob. Comput..

[35] Wang X., Chen G., Cao X., Zhang Z., Yang M., Jin S. (2022). Robust and Accurate Step Counting Based on Motion Mode Recognition for Pedestrian Indoor Positioning Using a Smartphone. IEEE Sens. J..

[36] Brajdic A., Harle R. (2013). Walk Detection and Step Counting on Unconstrained Smartphones.

[37] Gobana F.W. (2018). Survey of Inertial/Magnetic Sensors Based Pedestrian Dead Reckoning by Multi-Sensor Fusion Method.

[38] Weinberg H. Using the ADXL202 in Pedometer and Personal Navigation Applications. Vol. 2023-02-03, 2002. Online Resource. http://www.bdtic.com/DownLoad/ADI/AN-602.pdf.

[39] Tulapurkar H., Banerjee B., Buddhiraju K.M. (2023). Multi-head attention with CNN and wavelet for classification of hyperspectral image. Neural Comput. Appl..

[40] Ciresan D.C., Meier U., Masci J., Gambardella L.M., Schmidhuber J. Flexible, high performance convolutional neural networks for image classification. Proceedings of the Twenty-Second International Joint Conference on Artificial Intelligence.

[41] Cortes C., Vapnik V. (1995). Support-vector networks. Mach. Learn..

[42] Zhu J., Ju Y., Xia M. (2021). Vehicle recognition model based on improved CNN-SVM. Proceedings of the 2021 2nd International Seminar on Artificial Intelligence, Networking and Information Technology (AINIT).

[43] Wang Y., Cheng H., Meng M.Q.H. (2022). Inertial Odometry Using Hybrid Neural Network with Temporal Attention for Pedestrian Localization. IEEE Trans. Instrum. Meas..

[44] Wang Y., Rajamani R. (2018). Direction cosine matrix estimation with an inertial measurement unit. Mech. Syst. Signal Process..

[45] Shuster M.D., Oh S.D. (1981). Three-axis attitude determination from vector observations. J. Guid. Control..

[46] Hajati N., Rezaeizadeh A. (2021). A Wearable Pedestrian Localization and Gait Identification System Using Kalman Filtered Inertial Data. IEEE Trans. Instrum. Meas..

